# Non-invasive quantification of ^18^F-florbetaben with total-body EXPLORER PET

**DOI:** 10.21203/rs.3.rs-3764930/v1

**Published:** 2023-12-27

**Authors:** Emily N. Holy, Elizabeth Li, Anjan Bhattarai, Evan Fletcher, Evelyn R. Alfaro, Danielle J. Harvey, Benjamin A. Spencer, Simon R. Cherry, Charles S. DeCarli, Audrey P. Fan

**Affiliations:** 1 Department of Neurology, University of California (UC) Davis Health; 2 Department of Biomedical Engineering, UC Davis; 3 Department of Radiology, UC Davis Health; 4 Department of Public Health Sciences, UC Davis Health

**Keywords:** ^18^F-florbetaben, Alzheimer disease, β-Amyloid, total body EXPLORER PET, kinetic modeling, image derived input function

## Abstract

**Purpose:**

Kinetic modeling of ^18^F-florbetaben provides important quantification of brain amyloid deposition in research and clinical settings but its use is limited by the requirement of arterial blood data for quantitative PET. The total-body EXPLORER PET scanner supports the dynamic acquisition of a full human body simultaneously and permits noninvasive image-derived input functions (IDIFs) as an alternative to arterial blood sampling. This study quantified brain amyloid burden with kinetic modeling, leveraging dynamic ^18^F-florbetaben PET in aorta IDIFs and the brain in an elderly cohort.

**Methods:**

^18^F-florbetaben dynamic PET imaging was performed on the EXPLORER system with tracer injection (300 MBq) in 3 individuals with Alzheimer’s disease (AD), 3 with mild cognitive impairment, and 9 healthy controls. Image-derived input functions were extracted from the descending aorta with manual regions of interest based on the first 30 seconds after injection. Dynamic time-activity curves (TACs) for 110 minutes were fitted to the two-tissue compartment model (2TCM) using population-based metabolite corrected IDIFs to calculate total and specific distribution volumes (V_T_, V_s_) in key brain regions with early amyloid accumulation. Non-displaceable binding potential (*BP*_*ND*_) was also calculated from the multi-reference tissue model (MRTM).

**Results:**

Amyloid-positive (AD) patients showed the highest V_T_ and V_S_ in anterior cingulate, posterior cingulate, and precuneus, consistent with *BP*_*ND*_ analysis. *BP*_*ND*_ and V_T_ from kinetic models were correlated (r^2^ = 0.46, P<2*e*^−16^) with a stronger positive correlation observed in amyloid-positive participants, indicating reliable model fits with the IDIFs. V_T_ from 2TCM was highly correlated (*r*^2^ = 0.65, P< 2*e*^−16^) with Logan graphical V_T_ estimation.

**Conclusion:**

Non-invasive quantification of amyloid binding from total-body ^18^F-florbetaben PET data is feasible using aorta IDIFs with high agreement between kinetic distribution volume parameters compared to *BP*_*ND*_ in amyloid-positive and negative older individuals.

## Introduction

Amyloid imaging with PET radiotracers has proven useful for clinical assessment of Alzheimer’s disease and monitoring of response to recently developed anti-amyloid therapies [1,2]. Typical PET assessment of amyloid is either based on a clinical read (amyloid positive or negative); or quantifies standardized uptake value ratio (SUVR) with reference to a predetermined reference region [3,4]. However, SUVR does not consider potential confounding variables such as blood volume, tracer metabolism, blood flow, and other pharmacokinetic components representative of tracer dynamics. Furthermore, dynamic PET information of amyloid uptake reveals additional, complementary physiological information at various uptake times, e.g., with early-phase images reflective of tracer delivery via cerebral blood flow and late-phase images reflective of equilibrium amyloid binding [5,6].

Kinetic model-based approaches are a quantitative alternative to SUVR that characterize distinct tracer dynamics (e.g., blood flow) and account for various physiological processes. Current amyloid tracers such as ^18^F-florbetaben, a second-generation amyloid tracer with more specific binding and less off-target binding relative to the first generation, are typically modeled with two tissue compartments. Due to florbetaben’s reversibly binding nature, the tissue-to-plasma equilibrium ratio not only reflects the available specific binding site density but also free and nonspecific binding of tracer [7]. Non-displaceable binding potential (*BP*_*ND*_) is a typical measurement from reference tissue methods and can be measured without arterial plasma measurements and it describes the ratio at equilibrium of specifically bound tracer to that of non-displaceable radioligand in tissue. BP_ND_ measurements from dynamic PET also benefit from being less sensitive to changes in cerebral blood flow (CBF) and noise related to SUVR [8]. On the other hand, full compartment modeling with a two-tissue model directly estimates specific rate constants such as K_1_, which reflects both tracer delivery (perfusion) and extraction fraction, k_3_ reflecting metabolism or binding, clearance from the tissue back to the blood (k_2_, k_4_), and blood volume (*V*_*b*_). Additionally, parameters more directly related to receptor binding density can be calculated, including the total (V_T_) and specific distribution volumes (V_S_) [9]. While binding potential and the volume of distribution measures are expected to correlate [7], the kinetic rate parameters estimated from two-tissue compartment modeling add further insight into specific underlying processes that govern amyloid PET uptake over time.

However, one disadvantage of full kinetic modeling is the requirement of an arterial blood input function, which is typically measured through multiple arterial blood samples during the PET scan. Arterial cannulation blood sampling is invasive, potentially painful, time consuming, and often discourages patients from participating in clinical research involving dynamic PET imaging. Sampling errors can also occur, and the arterial input function needs to be “low-noise” to avoid error propagation to kinetic estimates [10]. In addition, there is an inherent tradeoff between spatial and temporal resolution to achieve adequate signal-to-noise of dynamic PET frames for robust kinetic parameter fits [11,12]. Using conventional PET scanners with ~ 20 cm field of view (FOV) only a limited FOV of continuous dynamic data is available and will suffer from multiple gaps in time if an image-derived input function (IDIF) is used. The uEXPLORER total-body PET system reduces these tradeoffs and allows for the acquisition of IDIFs from vessels and arteries in the body at early time frames to be used as a surrogate for arterial blood sampling while simultaneously acquiring dynamic brain activity. Moreover, the high sensitivity of the uEXPLORER scanner allows for fast temporal sampling, i.e., 2-second frames especially at early time points, while maintaining high image resolution, thus overcoming previous challenges with IDIF methods [11,13].

The purpose of this study is to leverage the total-body EXPLORER scanner to extract reliable IDIFs from the descending aorta and apply a kinetic modeling-based approach to quantify brain β-Amyloid in an elderly cohort. We used non-invasive two-tissue compartment modeling to quantify V_T_ and V_S_ across brain regions and evaluated them with *BP*_*ND*_ values derived from a reference tissue-based model. Additionally, microparameters (K_1_, k_2_, k_3_, and k_4_) were derived and evaluated. Furthermore, we expected to see that regional measures of V_T_ and V_S_ from kinetic modeling are elevated in “index” brain regions that has previously been shown to accumulate amyloid in amyloid-positive versus amyloid-negative individuals [14].

## Materials and Methods

### Subjects

We recruited 15 participants, including 3 participants with Alzheimer’s Disease (AD, 75.7 ±4.6 years), 3 with mild cognitive impairment (MCI, 84.5 ±10.6 years), and 9 healthy controls (HC, 77.2 ±6.0 years) from the UC Davis Alzheimer’s Disease Research Center cohort. All 3 AD patients were amyloid-positive (Aβ+), and all HC and MCI individuals were amyloid-negative (Aβ−), resulting in 3 amyloid-positive cases and 12 amyloid-negative cases. Eligibility criteria included: age over 65 years, able to undergo an MRI, and known cognitive status based on clinical assessment and neuropsychological testing. Individuals with pacemakers, brain tumors, alcoholism and/or those who were not able to lie still for 90 minutes were excluded from this study. The UC Davis Institutional Review Board approved this protocol and written informed consent was obtained for all subjects involved in the study.

### PET and MRI Acquisition

#### Amyloid-PET

PET data were acquired with the UC Davis total-body uEXPLORER PET scanner [12,15]. ^18^F-florbetaben was administered to 15 participants via bolus injection with an average dose of 277.1 ±22.4 MBq. An ultra-low dose CT was obtained for anatomic localization and attenuation correction purposes immediately before the dynamic PET acquisition. The 140 kVp ultra-low dose CT had a tube current of ~5 mAs with automatic dose modulation, leading to an estimated dose of 1 mSv per acquisition. CT images were reconstructed into a 1024×1024 matrix with a 2.344 mm slice thickness and 512 mm axial FOV. Dynamic PET data were acquired over 110 minutes and reconstructed into 30 × 2-second frames, 12 × 10-second frames, 7 × 1-minute frames, and 20 × 10-minute frames following a high-temporal resolution total-body PET protocol established previously [16]. All image data were reconstructed using time-of-flight based ordered-subset expectation-maximization (OSEM) with all standard corrections but without point spread function modeling. Four OSEM iterations of 20 subsets were employed. Images were reconstructed with a 2.344 mm isotropic voxel size and without post-reconstruction smoothing following the UC Davis clinical protocol [17].

Amyloid positivity was determined by a clinical read on static PET scans by a trained neurologist (C.D.) with over 30 years of experience and certified to clinically determine PET amyloid positivity.

#### MRI

All participants underwent MRI scans on a 3 Tesla Siemens Tim Trio whole-body scanner equipped with a 32-channel head coil. The scanning parameters of the T1-weighted 3D magnetization-prepared rapid gradient-echo (MPRAGE) sequence included: matrix size=240×256, in-plane spatial resolution=1mm, repetition time=2300 ms, echo time=2.98 ms, flip angle=9 degrees, acquisition time=9 min 14 s, inversion time=900–1100 s, and 176 sagittal slices with thickness=1.0–1.2 mm. PET and MRI scans were taken on average 2.5 ±1.5 years apart from each other.

### Image-Derived Input Function

**Figure 1.**
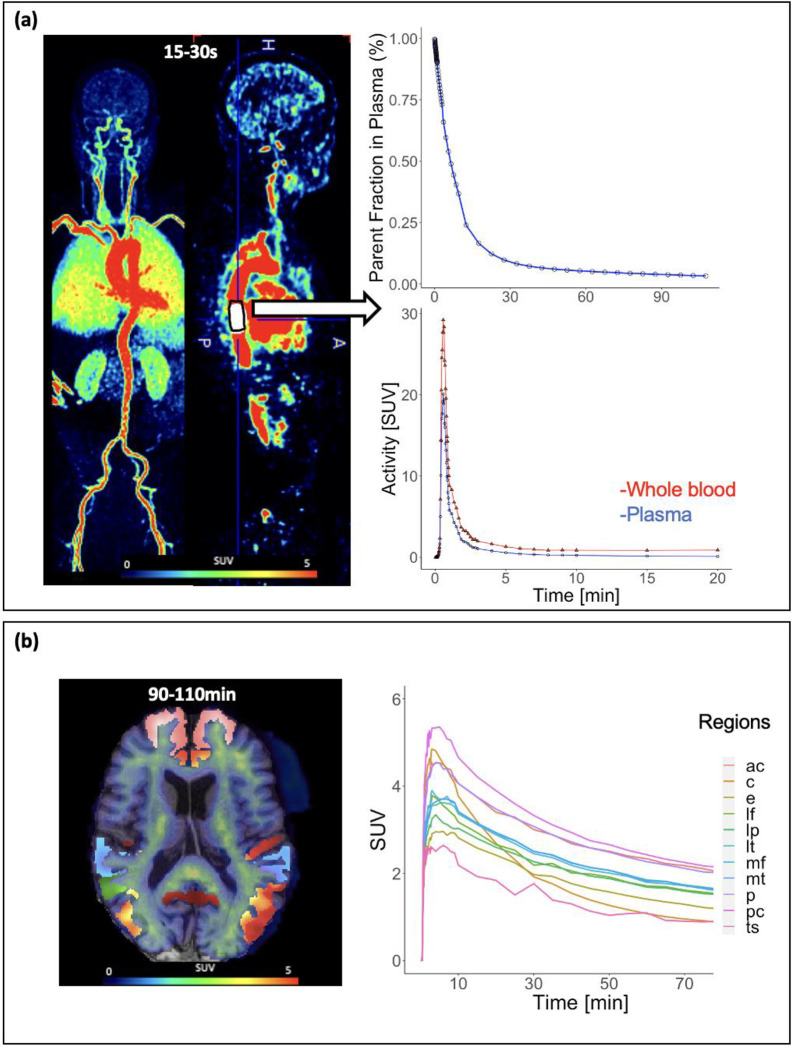
Dynamic total-body ^18^F-florbetaben data. (A) Literature-based metabolite fraction curve [21] (top), dynamic PET aorta image-derived input function representing whole blood(red) and plasma(blue)(middle). (B) Brain tissue time-activity curves (right) for a 73 year-old male with Alzheimer’s Disease. Anterior cingulate (ac), cerebellar gray matter (c), entorhinal cortex (e), lateral frontal cortex (lf), lateral parietal cortex (lp), lateral temporal cortex (lt), medial frontal cortex (mf), medial temporal cortex (mt), precuneus (p), posterior cingulate (pc), and temporal sulci (ts).

15 hand-drawn IDIFs were manually extracted in PMOD (PMOD Technologies, LLC) from the descending aorta from an early time window (mean initial frame 25.3s ±6.9s, mean volume = 19.4 ±5.2cm^3^) (Fig. 1). The IDIF region of interest (ROI) length was kept consistent across all individuals and was 18–20 voxels in the superior-inferior direction. IDIF ROI diameters varied to align with individual aorta anatomy but were eroded by 1 mm in all dimensions to avoid including the vessel walls. To completely avoid blood sampling, the IDIF was corrected for metabolite and plasma fractions using population-based curves from the literature prior to kinetic modeling. Plasma correction was applied by multiplying by a constant value of 0.73 for the whole blood: plasma ratio [18]. A bi-exponential function describing the fraction of unmetabolized ^18^F-florbetaben over time was then applied for metabolite correction derived from a previous study consisting of 10 patients with AD and 10 aged matched HCs [19].

#### Dynamic Processing

The dynamic PET data for each individual underwent brain extraction using PMOD and the FMRIB Software Library (FSL) brain extraction tool [20]. Dynamic PET motion correction was first performed frame by frame to an average image using FSL’s MCFLIRT, and linear (affine) registration was performed to align the 4D motion-corrected dynamic PET data with their respective T1-weighted (T1W) images using FSL’s FLIRT [22,23].

Using the Desikan-Killiany-Tourville (DKT) atlas, brain regions of interest that are known to accumulate amyloid were placed in the 10 following index regions: lateral frontal cortex (LF), medial frontal cortex (MF), anterior cingulate (AC), posterior cingulate (PC), lateral temporal cortex (LT), lateral parietal cortex (LP), medial temporal cortex (MT), entorhinal cortex (E), temporal sulci (TS), and the precuneus (P) by combining several subregions based on literature [3,24], (Supplemental Figure 1). These ROIs were obtained using subject-specific T1-weighted image segmentation with in-house software [24]. Time-activity curves were extracted from each of the brain regions and expressed as standardized uptake value (SUV, Fig. 1b). The cerebellar grey matter ROI was segmented using the DKT atlas described above for reference tissue-based modeling.

### Kinetic Modeling

#### Compartment Modeling

Kinetic modeling of regional time-activity curves was performed both using the 2-tissue compartment model (2TCM) and Logan graphical analysis. Kinetic modeling was performed using in-house software and specific methods used for quantification are described in detail in the literature [16,19]. For the 2TCM, delay between the aorta IDIF and brain time-activity curves (TACs) was calculated using joint estimation using all 69 reconstructed timepoints [25]. Results were obtained using a nonlinear least-squares fitting process. Initialization parameters are described in Supplemental Table 1.

Eq. 1 $$V_{S}=\frac{K_{1}k_{3}}{k_{2}k_{4}}$$

Eq. 2 $${\;V}_{T}=\frac{K_{1}}{k_{2}}(1+\frac{k_{3}}{k_{4}})$$

2TCM kinetic parameters such as, V_b_,K_1_,k_2_,k_3_,k_4_, specific distribution volume (V_S_, Eq. 1), and total distribution volume (V_T_, Eq. 2) were extracted independently for all 10 brain regions. V_b_ represents blood vlume fraction. K_1_ (mL/min/cm^3^) describes tracer transport from arterial plasma into the first tissue compartment, while k_2_ (1/min) describes transport from the first tissue compartment back into the blood pool. The k_3_ and k_4_ rate parameters (units 1/min) describe forward and backward transport, respectively, of the tracer between the two tissue compartments.


Eq. 4 $$LoganV_{T}=\frac{\int_{0}^{t}C_{T}(t)dt}{C_{T}(t)}=V_{T}\frac{\int_{0}^{t}C_{P}(t)dt}{C_{T}(t)}+Int$$

Logan graphical analysis was also performed to estimate total distribution volume (V_T_, Eq. 4) in MATLAB (version 2020a), using the image-based input function C_P_(t) and the brain time-activity curve, C_T_(t), for each ROI (^17^). t* was set to 30 minutes and V_T_ was calculated separately for each brain region.


Eq. 5 $$BP_{ND}=DVR-1=\frac{\left(V_{T}-V_{ND}\right)}{V_{ND}}$$

Multilinear reference tissue modeling (MRTM) was performed to quantify non-displaceable binding potential using cerebellar grey as the reference. t* was set to 30 minutes. (BP_ND_, Eq. 5).

### Statistical Analysis

Because measurements of the outcomes of interest (V_T_, V_S_, and BP_ND_) were available in several brain regions for each person, linear mixed effects models were used to understand how amyloid status and brain regions were associated with outcomes, including a person-specific random effect to account for multiple regions within a person. Separate models were fit for each outcome measure. Similar linear mixed effects models were also used to describe the effect of amyloid status on each kinetic rate parameter. Exploratory analysis was performed to investigate any driving microparameters behind V_T_ and V_S_ outcome measures using the same principles of the linear mixed effects models described above. Benjamini-Hochberg False discovery rate (FDR) was applied to correct for multiple comparisons when reporting p-values [26]. To assess the correlation between both V_T_ and V_s_ with BP_ND_, V_T_ with K_1,_ as well as to correlate V_T_ measures from the two-tissue compartment model versus Logan analysis, all measurements across regions were included. For these correlations, a linear mixed effects model was again used with a person-specific random effect, although in these models, brain region was not of specific interest and therefore not included as a variable in the model. All analyses were conducted using RStudio and a significance level of 0.05 was used [27].

## Results

### Compartment Models

**Figure 2.**
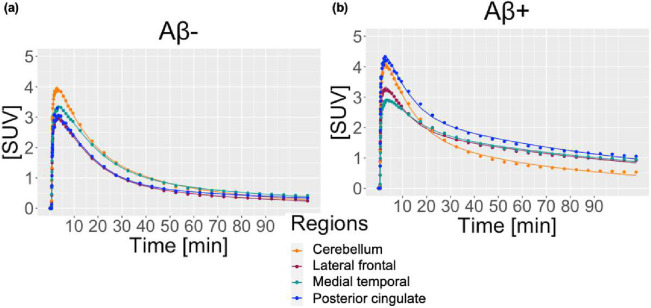
Two-tissue compartment model fits to measured time-activity curves for lateral frontal cortex, medial temporal cortex, posterior cingulate, and cerebellar gray matter in (A) 82 year-old male, cognitively normal participant and (B) 81-year old male patient with Alzheimer’s disease. IDIFs from the aorta were corrected for population-based metabolite fraction and plasma fraction.

High quality fits of β-Amyloid binding were achieved through the 2TCM model with the corrected IDIF and accounting for time delay through joint estimation. Typical time-activity curves for 1 HC/Aβ− and 1 patient with AD/Aβ+ are shown in Figure 2, revealing good model fits to our data. In the Aβ− individual, the lateral frontal, medial temporal and posterior cingulate showed similar TAC patterns as the cerebellar gray. The signal reached peaks (~3–4 SUV) for all regions and revealed no regional separation over later time points. In the Aβ+ patient, TAC peaks during the early time point were similar (~3–4 SUV) across all regions; however, unlike the Aβ− case, brain index regions showed higher SUV compared to cerebellar gray over time, especially after 30 minutes.

### Kinetic Modeling Quantification

**Figure 3.**
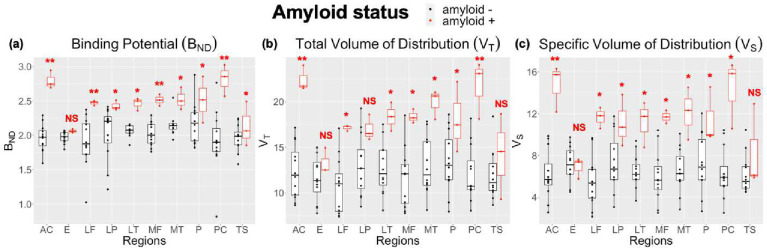
Regional BP_ND_ (A), V_T_ (B), and V_s_ (C) values for cortical regions: lateral frontal (LF), entorhinal (E), medial frontal (MF), temporal sulci (TS), lateral temporal (LT), precuneus (P), posterior cingulate (PC), and anterior cingulate (AC). Amyloid-positive individuals showed the highest values in all three parameters compared to amyloid-negative cohorts in relation to the interquartile range.

BP_ND_ was elevated across regions in Aβ+ participants (2.47 ±0.28) compared to Aβ− participants (1.14 ±0.17), which agreed with the visual clinical assessment. Similar average elevations in V_S_ (Aβ+ = 11.36 ±3.05; Aβ− = 6.48 ±2.26) and V_T_ (Aβ+ = 18.04 ±3.44; Aβ− = 12.24 ±3.01) were observed from 2TCM fitting (Fig. 3). Figure 3 reports the significant difference between Aβ+ and Aβ− groups within each region from mixed-effects modeling (i.e., where the region serves as its own reference in each interaction of the model). After applying this linear mixed-effects model to consider the interaction effect between amyloid status and brain region, FDR analysis revealed that the Aβ+ group had significantly higher BP_ND_, V_T_, and V_S_ in all index regions, except for the entorhinal cortex, lateral parietal, and temporal sulci. Multiple significant interactions between regions were also observed in all measures. The anterior cingulate and posterior cingulate showed the highest discrimination between amyloid positive and amyloid negative groups in all measures.

Table 1 lists kinetic rate parameters estimated by the 2TCM from three example cortical regions (lateral frontal, medial temporal and posterior cingulate) and the cerebellar gray reference from the Aβ+ and Aβ− groups. Identical but separate mixed effects models were used to describe statistical differences in V_T_, V_S_, and rate parameters. K_1_, k_2_, k_3_, k_4_, delay, and blood volume parameters values revealed no significant differences between the two amyloid status groups. No significant difference was observed in V_S_ or V_T_ for the cerebellar cortex between the Aβ+ and Aβ− groups as expected, indicating it is an appropriate reference. However, the Aβ+ showed significantly higher V_T_ and V_s_ values in all three index regions compared to Aβ− (P<0.05). Considering intragroup comparisons, within the Aβ+ group, V_T_ and V_S_ in all index regions were significantly higher than the same measures in cerebellar gray (P<0.05), while no significant difference was observed between regions within the Aβ− group.

### Regression Modeling of Model Parameters: Comparison of Amyloid Binding Parameters

**Figure 4.**
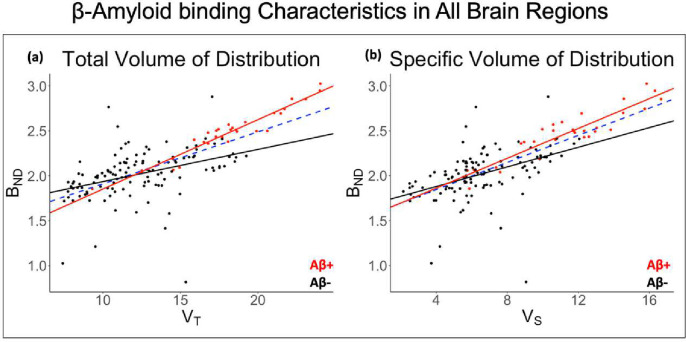
Linear regression analysis results accounting for subject clustering for Aβ+ (red), Aβ− (black), and all subjects (blue dashed). The following model statistics are for all subjects. (A) V_T_ and BND, (r^2^ = 0.46, P<2*e*^−16^). (B) V_S_ and BND, (r^2^ = 0.51, P<2*e*^−16^).

**Figure 5.**
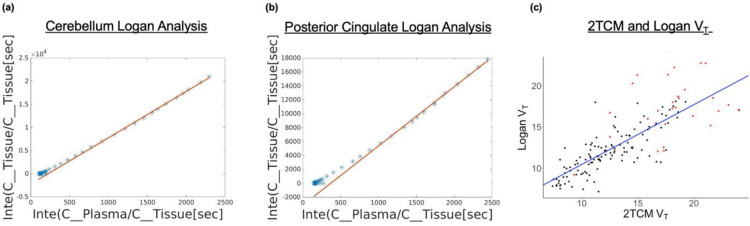
Examples of logan graphical analysis for one subject in the cerebellar reference region (A) and the Posterior Cingulate (B). Correlation between two-tissue compartment model and Logan analysis results for V_T_, accounting for subject clustering. V_T_ [2TCM] and V_T_ [Logan], (r^2^ = 0.65,). V_T_[Logan] = 0.73 V_T_ [2TCM] +3.15 (C).

Both V_S_ (P<2*e*^−16^) and V_T_ (P<2*e*^−16^) were highly correlated with BP_ND_ across participants (Fig. 4). Aβ+ individuals also demonstrated slight overestimation of BP_ND_ compared to both V_T_ and V_S_ values, with higher positive slope than Aβ− individuals. V_T_ , V_S_ , and BP_ND_ had no correlations (P>0.05) with K_1_ across all participants. However, separate K_1_ model statistics for the Aβ+ group and Aβ− group showed slight correlation with V_T_ (Aβ+: P=0.05, Aβ−: P=7.8*e*^−6^), V_S_ (Aβ+: P=0.04, Aβ−: P=1.9*e*^−5^), and BP_ND_ (Aβ+: P=0.04, Aβ−: P=0.01) (Supplemental Fig. 5). Similarly, no correlation was observed between both V_T_ and V_S_ with the other microparameters (k_2_,k_3_,k_4_). V_T_ from Logan analysis was directly compared to V_T_ from the 2TCM, with both measures using the same aorta IDIF as input (Fig. 5). Logan V_T_ was highly correlated with 2TCM V_T_ across brain regions (r^2^=0.65, P<2*e*^−16^).

## Discussion

In this paper, we evaluated the feasibility of non-invasive amyloid quantification using kinetic modeling and aorta-based IDIFs with total-body ^18^F-florbetaben dynamic PET. Aβ+ individuals showed elevation compared to Aβ− individuals in index brain regions for BP_ND_, V_S_, and V_T_ measures. Compartment modeling parameters derived from IDIFs were highly correlated with non-displaceable binding potential (BP_ND_) derived from the multi-reference tissue model, demonstrating quantitative discrimination between Aβ+ and Aβ− participants while contributing additional information about individual kinetic rate outcome parameters and macroparameters (V_T_ and V_S_).

Through kinetic modeling, we achieved V_T_ and V_S_ values that were comparable to Becker et al. and were able to quantify differences between amyloid positive versus amyloid negative cases [19]. In addition to significant differences between Aβ+ and Aβ− for V_T_ and V_s_ values across index regions, significant interactions were also identified, suggesting that the differences in these values between Aβ+ and Aβ− varied by region. The general pattern of differences across regions was similar across all measures (BP_ND_, V_T_, and V_s_). Of note, the anterior cingulate, posterior cingulate, and precuneus had the highest signal and most significant discrimination between the two groups (Aβ+ versus Aβ−) that persisted across all three measures of tracer distribution. This observation is consistent with the regions’ key role in the default mode network which has been shown to have early accumulation of amyloid [28].

Quantification of distribution volumes and separate kinetic rate parameters has the potential to better characterize amyloid burden and provides more information about tracer distribution [19]. V_T_ and V_S_ values quantify amyloid binding density with from rate parameters with absolute units, and our average model values (Frontal cortex: V_S_: Aβ+ = 11.66; Aβ− = 5.48; V_T_: Aβ+ = 17.13; Aβ− = 10.76; Cerebellum: V_T_: Aβ+ = 11.47; Aβ− = 11.32) were consistent with Becker et al. Table 1 (Frontal cortex: V_S_: Aβ+ = 5.85; Aβ− = 1.86; V_T_: Aβ+ = 13.7; Aβ− = 7.22; Cerebellum: V_T_: Aβ+ = 7.85; Aβ− = 5.36) [19].

From mixed-effects modeling of the parameter values in Table 1, no single kinetic rate parameter that explained the differences in V_S_ and V_T_ between Aβ+ and Aβ− groups was found. In particular, V_s_ and V_T_ did not correlate with K_1_ across all participants (Supplementary Fig. 3) in this cross-sectional study, which suggests that those measures were not heavily influenced by perfusion-related tracer delivery to specific brain regions. However, some differences were observed when considering interactions between kinetic rate parameters, which may reflect their relationships in Eqs. 1 and 2. Our non-invasive quantification of multiple kinetic parameters could be combined with longitudinal follow-up in future studies to investigate complex relationships over time between amyloid buildup and specific processes (e.g., reduced perfusion) in patients with cognitive decline [29].

Compared to SUVR, compartment modeling of amyloid enables estimation of total distribution volume without assuming a pseudo-equilibrium, and thus is likely more reliable across variable scan durations and amyloid loads. Because it reflects pseudo-equilibrium of ^18^F-florbetaben as a reversibly binding tracer, SUVR can slightly overestimate model-based values especially for amyloid-positive individuals [19]. As an alternative, incorporating a balance between model complexity and quantitative amyloid characterization may be preferred and is possible with Logan graphical analysis. We demonstrated high correlation between V_T_ from Logan analysis and 2TCM using the IDIF similar to Su et al., suggesting that Logan-based V_T_ is a suitable quantitative measure without extensive computational modeling [30]. The variations in V_T_ and V_S_ across our amyloid-negative control group may reflect true inter-individual biological variations, which may not be reflected in SUVR values. These variations should be investigated further in future studies alongside a larger cohort of amyloid-positive individuals.

One of the major foci of our study was to assess the feasibility of using a descending aorta IDIF as the input function for compartment modeling. Carotid arteries present an advantage when considering delay due to their proximity to brain tissues, however, are subject to major partial volume effects due to their size [13]. The aortic arch was not chosen due to the complex flow because of connecting arteries and greater cardiac motion that may have influenced modeling of tracer delivery [31]. Using the ascending portion compared to the descending portion of the aorta provided similar IDIF curves, however the descending portion typically demonstrated less curvature and was more easily visible for manual drawing in the sagittal slice. Variability in our IDIF peak and spread may have had some effect on K_1_ and k_2_ components during modeling and could reflect varying bolus volumes across participants. Our K_1_ was slightly higher than Becker et al. (0.2–0.25 vs 0.3–0.51 *mL.cm*^−*3*^.*min*^−*1*^*)*, however all other kinetic parameters were consistent. Differences in values could be explained by variability between study populations, or use of arterial sampling of the blood input function and high-performance liquid chromatography for the metabolite correction in Becker et al., instead of an IDIF and literature values for corrections of the input function in our study. Relatively higher tracer delivery, K_1_, could also be due to the higher temporal framing provided by the high sensitivity of EXPLORER (i.e., short 2-second framing compared to slower sampling in previous studies) [32]. For example, figure 7 from Volpi et al. demonstrates the effect of higher resolution 2s framing can lead to a higher peak for IDIFs and various regional TACs compared to lower resolution 10s framing [33].

In the present study, we measured quantitative amyloid in target and reference tissues, and the 2TCM was used to describe tracer kinetics in both regions. We observed superior fitting of all brain index regions and the reference region using 2TCM compared to the 1TCM (Supplemental Figure 2). Initially, 1TCM was used to describe the cerebellar gray reference based on the idea that the cerebellar region has been shown to have little to no amyloid binding and therefore would presumably have insignificant k_3_ and k_4_ components [3]. In our analysis, the 2TCM performed better in the cerebellum which may be due to a slow nonspecific compartment. Kinetic modeling may be more accurate in cases where the cerebellum is not appropriate as a reference region, such as in familial AD that exhibits the presence of β-amyloid plaques in the cerebellum [34]. Future work could compare quantitative parameters in alternative reference regions such as whole cerebellum, white matter brain stem/pons, whole brainstem, and eroded subcortical white matter, which have shown good correlations with the gold standard plasma input-based quantification [35].

High quality kinetics in this work were enabled by the uEXPLORER total-body PET/CT scanner which has a 194 cm axial field of view and a very high detection sensitivity [12]. Future work will emphasize development of total-body PET methods that aim to improve image quality and to reduce radiotracer dose if serial clinical amyloid scans are required; or leverage deep learning to achieve higher temporal sampling. Efficient computations with leading-edge estimation of delay can also be applied to amyloid PET kinetics in future work to generate parametric maps of quantitative amyloid binding [36]. Additionally, future work with total-body PET may look at dynamics of amyloid signal to analyze links across different organs in AD pathogenesis similar to other studies of cancer that have been done with uEXPLORER [37].

Comprehensive quantification of amyloid PET has many applications for future clinical use, especially in the context of improving early diagnosis, therapeutic intervention, and secondary prevention. Diagnostic decisions are often based on clinical reads of static SUVR, which does not reflect amyloid load as a continuous process. Absolute quantification can highlight specific pathological changes at early stages of AD, both in amyloid and perfusion, which has a direct impact on enrolling subjects and establishing an optimal window for therapeutic intervention. In addition, quantification is used to monitor treatment in clinical trials; has been shown to change diagnosis, patient management, and predict cognitive decline [38]; and is enabled by non-invasive methodology as presented in this work.

There were several limitations to this study. Metabolite fraction correction in the present study was based on a population average from Patt et al. to avoid invasive blood sampling [21]. The true metabolite fraction estimation of our cohort may differ in this study despite similarities in the cohort of subjects. Individualized radiometabolite correction remains an unsolved challenge. Although previous studies have attempted to derive metabolite-free arterial input functions through the simultaneous estimation method (SIME) [39], estimation of tracer metabolism from multiple organ kinetics in a dynamic whole-body scan could be a future direction [40,41]. Total-body imaging uEXPLORER, may allow for development of whole-body physiological model of radiotracer metabolism, which would allow more accurate estimation of IDIF without needing information from separate populations or arterial sampling [33]. In addition, the number of subjects included in this initial study was relatively small, which we mitigate by leveraging the large amount of kinetic information across multiple brain regions in mixed-effects models. Our small sample with relatively few amyloid-positive cases made it difficult to directly evaluate the quantitative outcome measures (V_T_, V_s_) with BP_ND_, which will be addressed with larger samples in future studies. As our focus was amyloid quantification with IDIF, reference-tissue based kinetic modeling (e.g., estimation of distribution volume ratios) were outside the scope of the present study but may be included in future work as another amyloid measure [19].

## Conclusion

The total-body EXPLORER PET allows for high quality, dynamic kinetic modeling of the whole body. Absolute quantification of β-Amyloid and multiple kinetic rate parameters from total-body ^18^F-florbetaben dynamic PET is feasible using a descending aorta IDIF. These two aspects enable non-invasive acquisition of accurate and quantitative measures of amyloid accumulation in clinical research of aging and dementia.

## Figures and Tables

**Table 1.** Parameter values from Aβ+ and Aβ− individuals who underwent dynamic ^18^F-Florbetaben studies and subsequent kinetic modeling. AD individuals are included in the Aβ+ columns and both MCI and HC individuals are in Aβ− columns. The cortical areas or index regions were included due to their involvement in early Braak staging. These include the lateral frontal cortex (LF), medial temporal cortex (MT), and posterior cingulate (PC), with cerebellar gray matter(C) as the reference.

	Aβ+(n=3)				Aβ− (n=12)			
	Index Regions		Reference		Index Regions		Reference	
Regions	LF	MT	PC	C	LF	MT	PC	C
$K_{1}$ *(mL.cm ^−3^.min^−1^)*	0.480 ± 0.083	0.505 ± 0.071	0.570 ± 0.082	0.629 ± 0.065	0.405 ± 0.087	0.358 ± 0.064	0.520 ± 0.102	0.498 ± 0.097
$k_{2}$ *(min^−1^)*	0.094 ± 0.020	0.082 ± 0.018	0.102 ± 0.021	0.112 ± 0.027	0.076 ± 0.023	0.051 ± 0.020	0.071 ± 0.023	0.078 ± 0.021
$k_{3}$ *(min^−1^)*	0.019 ± 0.008	0.022 ± 0.011	0.021 ± 0.008	0.044 ± 0.043	0.040 ± 0.008	0.024 ± 0.015	0.033 ± 0.008	0.019 ± 0.006
$k_{4}$ *(min^−1^)*	0.019 ± 0.005	0.023 ± 0.019	0.021 ± 0.010	0.042 ± 0.028	0.018 ± 0.002	0.014 ± 0.003	0.018 ± 0.001	0.024 ± 0.003
V_T_ *(cortex)* V_T_ ’*(cerebellum) (mL.cm^−3^)*	**17.126 ± 0.300** * †	**19.905 ± 1.607** * †	**21.737 ± 3.181** * †	11.467 ± 0.268	10.761 ± 3.098	12.941 ± 3.110	12.071 ± 3.033	11.328 ± 1.941
Vₛ = “K₁.k₃” /“k₂.k₄” *(mL.cm^−3^)*	**11.661 ± 1.010** * †	**12.118 ± 2.513** * †	**14.360 ± 3.297** * †	5.038 ± 0.757	5.481 ± 2.405	6.508 ± 2.078	6.332 ± 2.402	5.403 ± 0.965
$V_{b}\left(\frac{mL}{mL}\right)$	0.040 ± 0.016	0.049 ± 0.037	0.052 ± 0.017	0.052 ± 0.017	0.042 ± 0.014	0.044 ± 0.011	0.056 ± 0.012	0.057 ± 0.018
*Delay (s)*	6.333 ± 1.528	6 ± 1	6.667 ± 1.528	7 ± 1	5.417 ± 1.730	5.417 ± 1.621	5.417 ± 1.929	6.083 ± 1.165

**Significance was found for the following:**

* For V_T_ and *V*_*s*_ Aβ+ index regions were significantly higher than Aβ− index regions *p<0.05

† Within the Aβ+ group index regions were significantly higher than cerebellar reference *p<0.05

## Data Availability

The datasets generated during and analysed during the current study are available from the corresponding author on reasonable request.
